# All Domains of SARS-CoV-2 nsp1 Determine Translational Shutoff and Cytotoxicity of the Protein

**DOI:** 10.1128/jvi.01865-22

**Published:** 2023-02-27

**Authors:** Ilya Frolov, Tatiana Agback, Oksana Palchevska, Francisco Dominguez, Alexander Lomzov, Peter Agback, Elena I. Frolova

**Affiliations:** a Department of Microbiology, University of Alabama at Birmingham, Birmingham, Alabama, USA; b Department of Molecular Sciences, Swedish University of Agricultural Sciences, Uppsala, Sweden; c Laboratory of Structural Biology, Institute of Chemical Biology and Fundamental Medicine SB RAS, Novosibirsk, Russia; Loyola University Chicago - Health Sciences Campus

**Keywords:** SARS-CoV-2, attenuating mutations, coronavirus, cytotoxicity, nsp1, nuclear magnetic resonance, protein structure-function, translation inhibition

## Abstract

Replication of the severe acute respiratory syndrome coronavirus 2 (SARS-CoV-2) strongly affects cellular metabolism and results in rapid development of the cytopathic effect (CPE). The hallmarks of virus-induced modifications are inhibition of translation of cellular mRNAs and redirection of the cellular translational machinery to the synthesis of virus-specific proteins. The multifunctional nonstructural protein 1 (nsp1) of SARS-CoV-2 is a major virulence factor and a key contributor to the development of translational shutoff. In this study, we applied a wide range of virological and structural approaches to further analyze nsp1 functions. The expression of this protein alone was found to be sufficient to cause CPE. However, we selected several nsp1 mutants exhibiting noncytopathic phenotypes. The attenuating mutations were detected in three clusters, located in the C-terminal helices, in one of the loops of the structured domain and in the junction of the disordered and structured fragment of nsp1. NMR-based analysis of the wild type nsp1 and its mutants did not confirm the existence of a stable β5-strand that was proposed by the X-ray structure. In solution, this protein appears to be present in a dynamic conformation, which is required for its functions in CPE development and viral replication. The NMR data also suggest a dynamic interaction between the N-terminal and C-terminal domains. The identified nsp1 mutations make this protein noncytotoxic and incapable of inducing translational shutoff, but they do not result in deleterious effects on viral cytopathogenicity.

**IMPORTANCE** The nsp1 of SARS-CoV-2 is a multifunctional protein that modifies the intracellular environment for the needs of viral replication. It is responsible for the development of translational shutoff, and its expression alone is sufficient to cause a cytopathic effect (CPE). In this study, we selected a wide range of nsp1 mutants exhibiting noncytopathic phenotypes. The attenuating mutations, clustered in three different fragments of nsp1, were extensively characterized via virological and structural methods. Our data strongly suggest interactions between the nsp1 domains, which are required for the protein’s functions in CPE development. Most of the mutations made nsp1 noncytotoxic and incapable of inducing translational shutoff. Most of them did not affect the viability of the viruses, but they did decrease the rates of replication in cells competent in type I IFN induction and signaling. These mutations, and their combinations, in particular, can be used for the development of SARS-CoV-2 variants with attenuated phenotypes.

## INTRODUCTION

Severe acute respiratory syndrome coronavirus 2 (SARS-CoV-2) has recently emerged, and, within a few months, it developed a worldwide pandemic of a highly debilitating COVID-19 disease (1–4). The characteristic features of SARS-CoV-2 include an exceptionally efficient human-to-human transmission and a high pathogenicity with significant rates of lethal outcomes. SARS-CoV-2 represents an unquestionable public health threat, but since it was discovered less than 3 years ago, our understanding of many aspects of its biology remains far from complete. We are likely only in the beginning stages of understanding the molecular mechanisms of SARS-CoV-2 replication and pathogenesis.

SARS-CoV-2 is one of the β-coronaviruses (β-CoVs) in the Coronaviridae family (3). Its spherical, enveloped virions contain a single-stranded genomic RNA (G RNA) of positive polarity of approximately 30 kb (5–7). Upon delivery into cells, it serves as a template for translation of viral nonstructural proteins (nsp1 to nsp16), which are required for synthesis of virus-specific RNAs and for modification of the intracellular environment. The nsps are translated as 2 polyproteins that are encoded by the overlapping open reading frames (ORF)1a and ORF1ab, and they are then processed into individual proteins by viral proteases. Structural and accessory proteins are individually translated from 8 subgenomic RNAs that are encoded by the 3′-terminal fragment of the G RNA.

SARS-CoV-2 is highly cytopathic, and its replication causes cell death within 1 to 2 days post infection (p.i.) (8). As in many other viral infections, the development of cytopathic effect (CPE) is likely a multicomponent process. However, previous studies suggested that, as in cases of other β-CoVs, SARS-CoV-2-specific nonstructural protein 1 (nsp1) is an important contributor to CPE and is one of the major virulence factors (9, 10). Thus far, nsp1 proteins of the β-CoVs have been shown to be multifunctional (9, 10) and directly responsible for (i) inhibition of cellular mRNA translation, (ii) redirection of the translational machinery to viral RNA templates, (iii) induction of cell cycle arrest in G_0_/G_1_ phase, and (iv) degradation of cellular messenger RNAs. They were also proposed as key contributors to downregulation of the innate immune response. In addition, nsp1 was suggested as a regulator of expression of virus-specific genes. Thus, nsp1 is involved in modification of the intracellular environment and, ultimately, in regulation of viral replication.

Inhibition of cellular RNA translation appears to be one of the major direct functions of nsp1. It is determined by at least two activities: the ability of the proposed C-terminal short α-helices to directly interact with the mRNA channel of 40S ribosomal subunit (11, 12) and the protein’s involvement in endoribonuclease activity that mediates degradation of cellular, but not viral, mRNA templates (13–16). The experimental data strongly suggest that nsp1 does not directly perform RNA cleavage but rather recruits yet unknown cellular or viral protein(s) for this function (16). Interaction of nsp1 with the 5′-terminal stem-loop structure in the β-CoV leader RNA sequence was proposed to drive selective translation of viral RNAs in the setting of virus-induced translational shutoff (17, 18).

β-CoV nsp1s are relatively short (approximately 200 aa long) proteins. They demonstrate low conservation between members of different β-CoV lineages, but remain conserved within species of the same lineage. The 3D structures of folded N-terminal domains have been resolved for nsp1 proteins of both SARS-CoV (aa 10 to 126) (19) and SARS-CoV-2 (aa 12 to 127) (20, 21). The endoribonuclease activity and interaction with the 5′UTR of virus-specific templates have been ascribed to this structured domain. The following C-terminal domain (aa 125 to 180) is intrinsically disordered in solution (22). Upon binding to the 40S ribosomal subunit, the most C-terminal fragment of nsp1 (aa 153 to 179) folds into two α-helices, which occupy the ribosomal mRNA-binding site (11, 12). This interaction appears to block binding of cellular mRNAs. Thus, it plays a critical role in the inhibition of translation. Some mutations in the C-terminal α-helices have deleterious effects on nsp1-induced translational shutoff (10). However, thus far, the available to date multiple cryoelectron microscopy (CryoEM) structures of the nsp1-ribosome complexes have not conclusively determined the position of the folded N-terminal domain on the 40S ribosomal subunit (11, 12). This suggests that the protein fragment upstream of the two C-terminal α-helices either does not interact with the ribosome or mediates only weak additional interaction(s).

In this study, we have shown that nsp1 alone can induce CPE. Next, we used an unbiased approach to select nsp1 mutants that lacked the ability to cause CPE. The identified mutations accumulated in three different fragments of the protein, including the N-terminal structured domain, the C-terminal α-helices, and the connecting disordered protein fragment. We have demonstrated that mutations in all three sites affected the ability of nsp1 to inhibit translation and induce CPE. In a viral context, the tested mutations had no effects on viral replication in cells that were deficient in type I IFN signaling and did not prevent virus-induced CPE. We also used nuclear magnetic resonance (NMR) to analyze the effects of two representative mutations on the structure of nsp1 and identified two structural elements, other than the C-terminal α-helixes, that are important for the inhibition of cellular translation.

## RESULTS

### The full-length SARS-CoV-2 nsp1, but not its structural elements, is cytotoxic.

The experimental dissection of nsp1 functions is relatively complicated, as its expression from most of the commonly used DNA cassettes has a feedback mechanism. Accordingly, the synthesized protein can inhibit its own expression by downregulating translation of the nsp1-coding mRNAs. In addition, the plasmid-based expression requires a high efficiency of DNA transfection that cannot be achieved in many cell types. Therefore, to analyze the effects of the SARS-CoV-2 nsp1 in vertebrate cells, we applied a set of alphavirus-based replicons (23, 24) that could mediate either transient or stable expression of the protein of interest.

**FIG 1:**
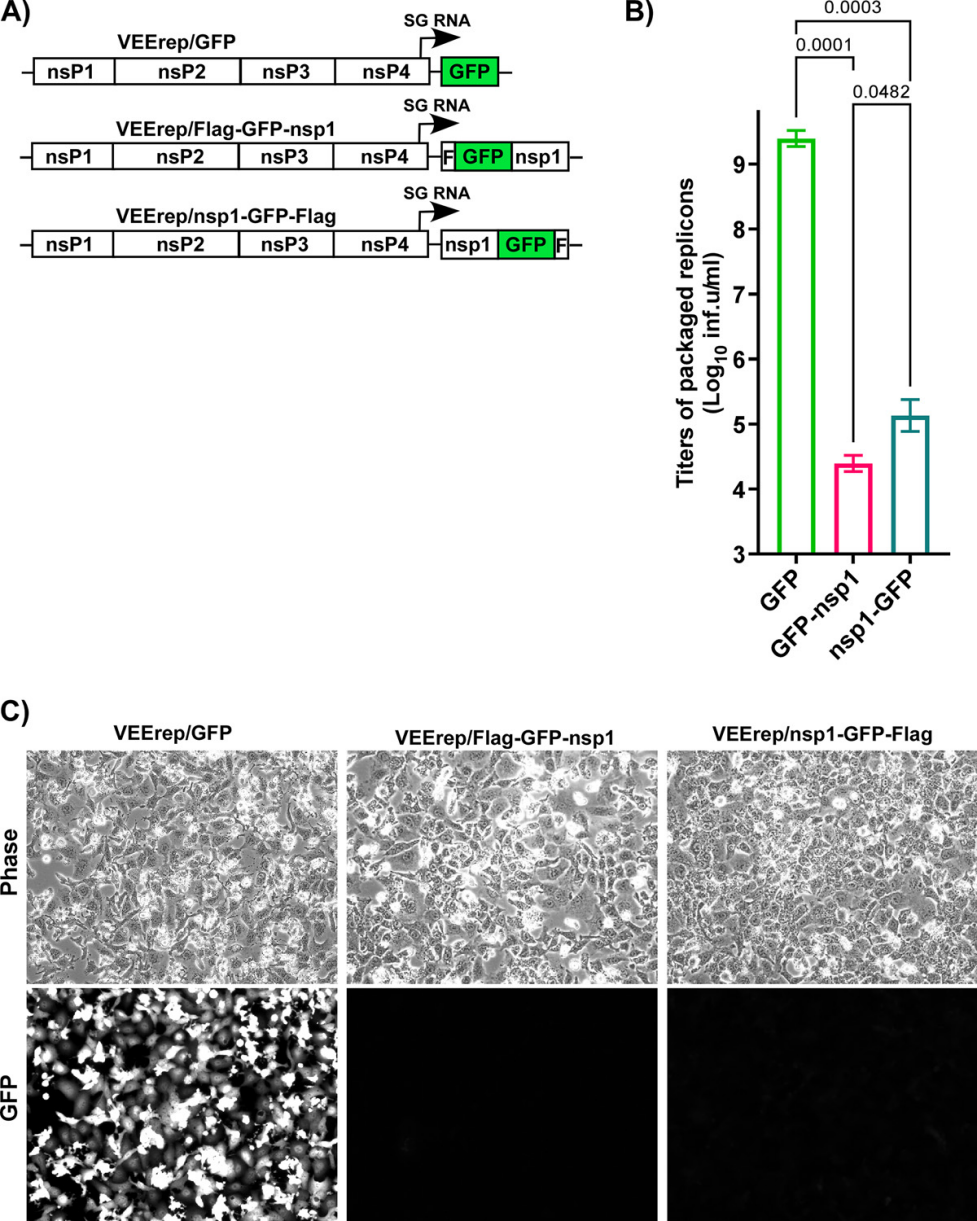
Expression of SARS-CoV-2 nsp1 has strong negative effects on replication of alphavirus replicons and their packaging into infectious virions. (A) The schematic representation of VEEV replicons encoding GFP, Flag-GFP-nsp1, and nsp1-GFP-Flag. (B) Equal amounts of *in vitro*-synthesized replicon and helper RNAs were coelectroporated into BHK-21 cells, as described in Materials and Methods. Viral particles were harvested at 24 h post electroporation, and their infectious titers were determined on BHK-21 cells. (C) Images of coelectroporated cells were acquired on a Nikon Eclipse fluorescence microscope before harvesting the media. The means and SDs are presented. The statistical significance of differences was determined by one-way ANOVA with a *post hoc* Dunnett’s multiple-comparison test (*n* = 3).

First, we cloned the nsp1-coding sequences as fusions with green fluorescent protein (GFP) into the wild type (wt) replicon of the Venezuelan equine encephalitis virus (VEErep) under control of the subgenomic (SG) promoter. GFP-Flag and Flag-GFP tags were fused with either the C or the N termini of nsp1 (VEErep/nsp1-GFP-Flag and VEErep/Flag-GFP-nsp1, respectively) (Fig. 1A). However, the attempts to package these constructs into infectious viral particles (see Materials and Methods for details) were unsuccessful (Fig. 1B). Electroporation of the *in vitro*-synthesized replicons’ and helper RNAs into BHK-21 cells caused cell death, but the titers of packaged replicons in the harvested stocks were 4 to 5 orders of magnitude below average levels, which are 2 to 5 × 10^9^ infectious units (inf.u)/mL (see VEErep/GFP in Fig. 1B as an example). In contrast to the control replicon expressing GFP only (VEErep/GFP), GFP expression from these replicons was almost undetectable (Fig. 1C). The most plausible explanation for such inefficient packaging and such low levels of expression of GFP-nsp1 fusions was that nsp1-mediated translation inhibition prevented accumulation of VEEV-specific nonstructural and structural proteins that is required for amplification of replicon and helper RNAs as well as for formation of viral particles. A conclusion from these experiments was that the expressed nsp1 fused with GFP retained its translation inhibitory functions and, thus, was cytotoxic.

**FIG 2:**
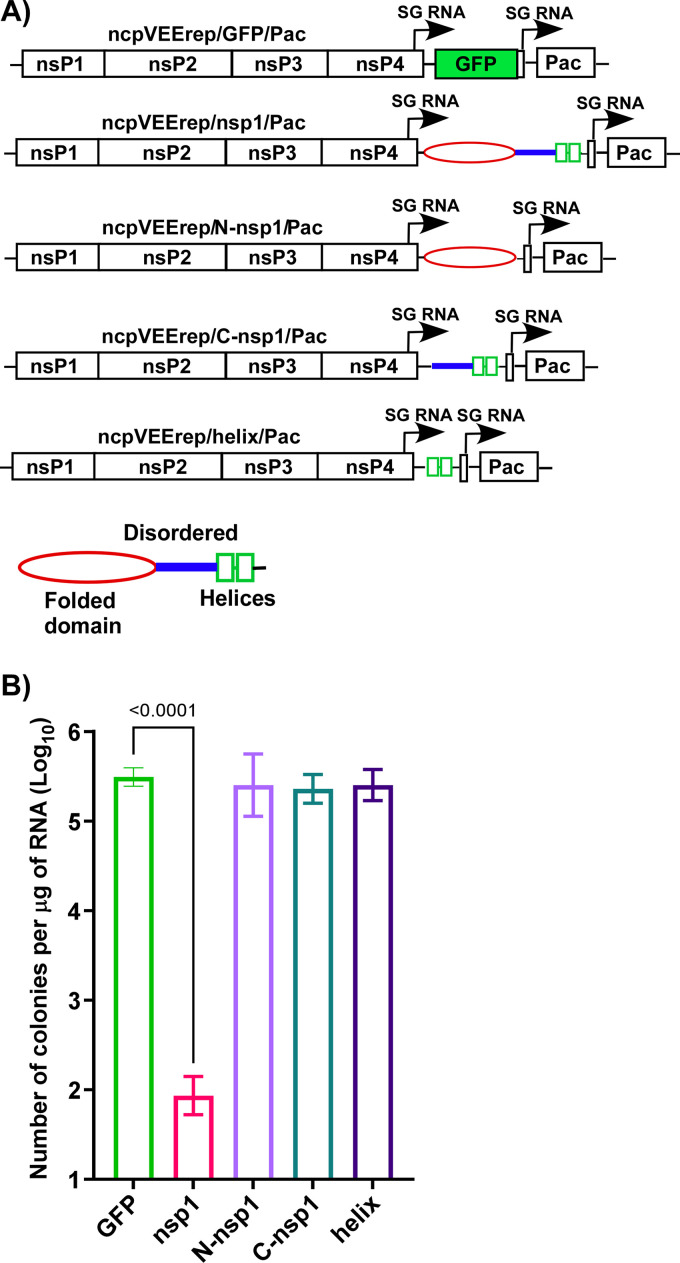
Expression of the entire SARS-CoV-2 nsp1, but not its fragments, is cytotoxic. (A) The schematic representation of noncytopathic VEEV replicons used in this study and the structural elements of SARS-CoV-2 nsp1. (B) Equal amounts of the *in vitro*-synthesized replicons’ RNAs were electroporated into BHK-21 cells, and colonies of Pur^R^ cells were selected, as described in Materials and Methods. The means and SDs are presented. The statistical significance of differences was determined by one-way ANOVA with a *post hoc* Dunnett’s multiple-comparison test (*n* = 3). Only the statistically significant differences are indicated.

Based on the above results, we changed the experimental system and applied an approach that we had previously developed for identification of functional elements in other cytotoxic viral proteins (24–28). Sequences encoding either the entire SARS-CoV-2 nsp1 or its structural domains were cloned into a mutated, noncytopathic VEEV replicon (ncpVEErep) under control of the SG promoter (Fig. 2A). The second promoter controlled expression of puromycin acetyltransferase (Pac), which makes cells resistant to translational arrest caused by puromycin (Pur) (Fig. 2A). In our experiments, Pur selection was used to eliminate the cells that did not receive replicons during electroporation. The designed replicons encoded (i) the full-length wt nsp1 (ncpVEErep/nsp1/Pac); (ii) its folded N-terminal domain (ncpVEErep/N-nsp1/Pac); (iii) the last half of the C-terminal domain (aa 146 to 180), which has been shown to interact with the 40S ribosomal subunit through two α-helices (ncpVEErep/helix/Pac); and (iv) the entire C-terminal domain (aa 125 to 180) of nsp1 (ncpVEErep/C-nsp1/Pac) (Fig. 2A). The ncpVEErep/GFP/Pac was used as a noncytopathic control. Equal amounts of the *in vitro*-synthesized RNAs were electroporated into BHK-21 cells (see Materials and Methods for details), and Pur selection was applied the next day. BHK-21 cells were used because they are efficiently transfected using RNA electroporations and are deficient in type I IFN signaling, which allows ncpVEErep replicons to readily establish persistent replication. At day 6 post electroporation, we evaluated the number of colonies of Pur^R^ cells (Fig. 2B). For ncpVEErep/GFP/Pac, essentially every transfected cell produced a colony of Pur^R^ cells. Cells electroporated with ncpVEErep/nsp1/Pac no longer divided, and the majority of them died within 3 to 4 days of Pur selection. They formed colonies with efficiencies that were orders of magnitude lower than those electroporated with ncpVEErep/GFP/Pac. However, replicons expressing individual domains and fragments of nsp1 induced the formation of colonies as efficiently as did the GFP-encoding construct. The results clearly demonstrated that the nsp1-induced cytotoxic effect requires nsp1 in its entirety and that the expression of its individual fragments does not result in cell death.

### Point mutations make nsp1 noncytotoxic.

In the above-described RNA transfection experiments, VEErep/nsp1/Pac induced very inefficient formation of Pur^R^ cell colonies, but a few colonies formed reproducibly (Fig. 2B). Their growth suggested a possibility that in the Pur^R^ cells, replicons expressed mutated and no longer cytopathic variants of SARS-CoV-2-specific nsp1. We randomly selected 12 colonies and sequenced the nsp1 genes in the persisting replicons. Most of the mutations abrogated the open reading frame (ORF) at different sites of nsp1 (data not shown). Only one cell clone contained a replicon with a single amino acid (L177P) substitution in the C terminus of nsp1, and this was at the end of the predicted short α-helix (11). This was likely responsible for the protein’s inability to inhibit translation of cellular mRNA templates and made cells capable of colony formation.

**FIG 3:**
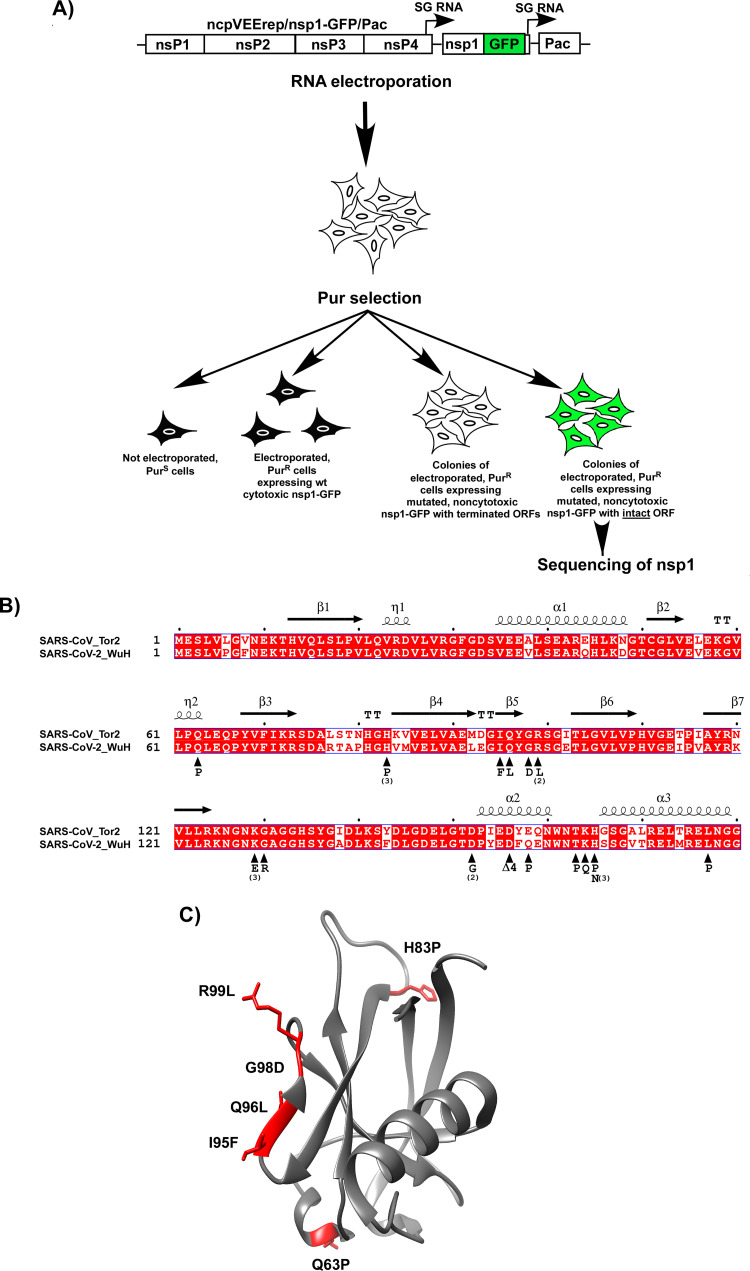
Point mutations in different fragments of SARS-CoV-2-specific nsp1-GFP fusion affect cytotoxicity of the protein. (A) The schematic representation of the protocol for selection of cell colonies containing VEEV replicons encoding noncytotoxic SARS-CoV-2 nsp1 mutants (see Materials and Methods for details). Colonies of GFP-positive Pur^R^ cells were used for identification of mutations in nsp1. (B) Alignment of SARS-CoV and SARS-CoV-2 proteins and distribution of the identified mutations. Numbers indicate the numbers of cells clones in which nsp1 contained the indicated mutations. Δ4 indicates the in-frame deletion of 4 amino acids. (C) The crystal structure (PDB ID: 7k7p) of the folded domain of the SARS-CoV-2-specific nsp1 and positions of the identified mutations.

Since the nsp1 variants with prematurely terminated ORFs were not informative in terms of identifying the amino acids that were important for protein’s function in CPE development, the experimental system was additionally modified. We took advantage of the low fidelities of both SP6 RNA polymerase, which was used for the *in vitro* RNA transcription, and alphavirus RdRp, and designed ncpVEErep/nsp1-GFP/Pac. Instead of nsp1, it encoded nsp1-GFP fusion under the control of an SG promoter (Fig. 3A). The formation of GFP-positive Pur^R^ cell colonies was expected to indicate the expression of noncytotoxic nsp1-GFP mutants with intact ORFs continuing into the GFP gene.

After the electroporation of the *in vitro*-synthesized ncpVEErep/nsp1-GFP/Pac RNA into BHK-21 cells, followed by Pur selection, most of the cells no longer divided and died within the next 3 to 4 days (Fig. 3A). However, some replicons expressed mutated, noncytotoxic nsp1, and, after 6 days of selection, we detected formation of colonies of Pur^R^ cells. Most of them were GFP-negative, but, among them, we readily identified the GFP-positive cell clones. The experiment was repeated 4 times, and GFP-positive colonies from different experiments were randomly selected for sequencing of the SARS-CoV-2 nsp1 gene. The identified mutations are presented in Fig. 3B and C. Some of them were detected in the replicons that were present in more than one colony. This was an indication that these amino acids are likely playing critical roles in nsp1-induced CPE. Importantly, most of the identified mutations clustered in three regions. In good agreement with previously published data, several of them were found in the C-terminal fragment (aa 155 to 180), which forms α-helices upon binding to the 40S ribosomal subunit (11). Thus, our selection system facilitated the unbiased identification of the mutations that made nsp1 noncytotoxic. Other mutations were found in the folded N-terminal domain, and two mutations were identified in the beginning of the intrinsically disordered C-terminal fragment. Interestingly, a cluster of 4 mutations was found in the fragment between amino acids 94 and 100 (Fig. 3B and C). This peptide is identical in SARS-CoV and SARS-CoV-2-specific nsp1 proteins, but it has been proposed to acquire different secondary structures (19–21). Taken together, these data provide additional support to the above conclusion that the nsp1-specific CPE is complex and is likely determined by the entire protein, rather than by its individual fragments.

### Nsp1 variants with identified mutations do not induce CPE.

**FIG 4:**
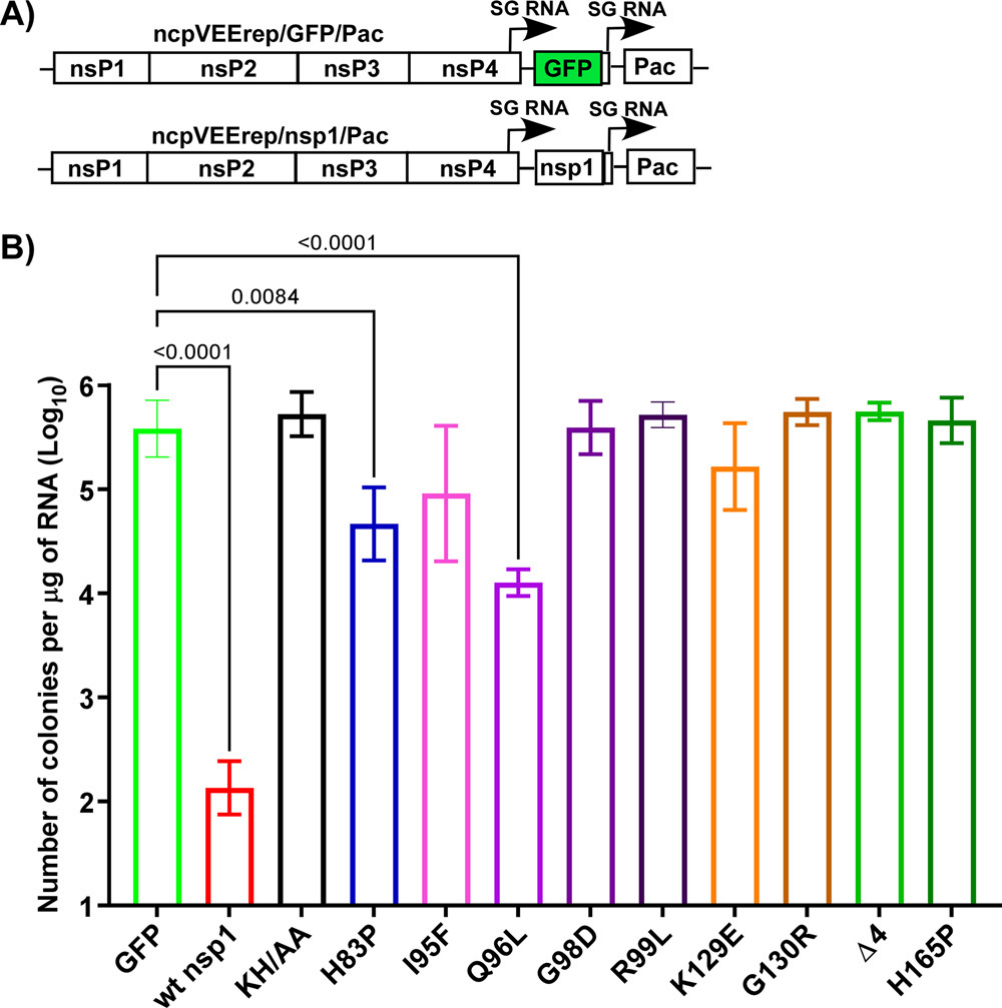
The identified point mutations affect cytotoxicity of SARS-CoV-2 nsp1. (A) The schematic representation of VEEV replicons encoding nsp1 or GFP. (B) The indicated mutations were cloned into the nsp1-coding sequence of ncpVEErep/nsp1/Pac. Equal amounts of the *in vitro*-synthesized RNAs were electroporated into BHK-21 cells, and colonies of Pur^R^ cells were selected, as described in Materials and Methods. The efficiencies of colony formation by mutated replicons were compared to those of parental ncpVEErep/nsp1/Pac and ncpVEErep/GFP/Pac. The means and SDs are presented. The statistical significance of differences was determined by one-way ANOVA with a *post hoc* Dunnett’s multiple-comparison test (*n* = 3). Only the statistically significant differences are indicated.

To confirm the attenuating effects of the identified mutations, we introduced some of them, including the in-frame deletion of 4 amino acids and the previously described K164A+H165A (KH/AA) (11, 16), into the nsp1 of ncpVEErep/nsp1/Pac (Fig. 4A). The noncytotoxic phenotype of the expressed protein was evaluated by transfecting the *in vitro*-synthesized replicon RNAs into BHK-21 cells, followed by Pur selection and assessment of the efficiencies of colony formation. Most of the tested mutations made nsp1 noncytotoxic, and the efficiencies of Pur^R^ replicon-containing colony formation were the same as that of the control VEErep/GFP/Pac (Fig. 4B). Some of the mutations, such as H83P and Q96L reduced the cytotoxic effect of nsp1 but did not eliminate it completely. Cells containing these replicons divided slower and had abnormal morphologies. Thus, the noncytopathic phenotype was clearly determined by the mutations that were introduced into the ribosome-binding C-terminal α-helices, including the previously described KH/AA. However, some mutations in the structured N-terminal domain, as well as K129E and G130R substitutions in the disordered fragment, were also sufficient to make nsp1 noncytotoxic.

### Identified mutations make nsp1 incapable of inhibiting cellular translation.

**FIG 5:**
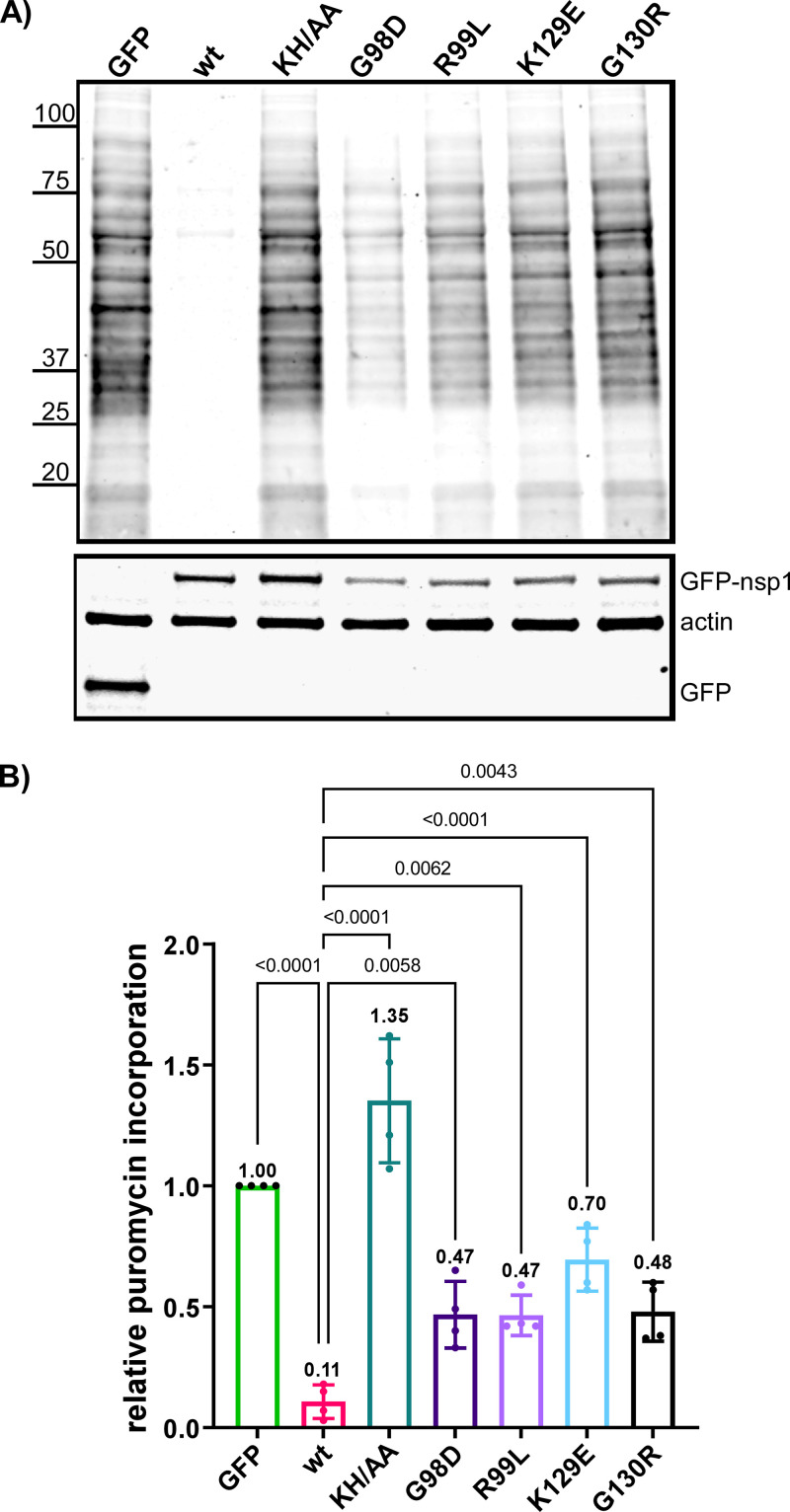
The identified mutations affect nsp1-induced inhibition of cellular translation. (A) BHK-21 cells were transfected with plasmids encoding GFP-fusions with either wt nsp1 or its variants with the indicated mutations. At 18 h post transfection, cells were incubated for 15 min in the media supplemented with 10 μg/mL of Pur. Cell lysates were processed, as described in Materials and Methods. The top panel presents a Western blot stained with anti-Pur and corresponding secondary antibodies. The images were acquired on a LI-COR imager. (B) Quantitation of the Pur signal of the blots, one of which is presented in panel A. The means and SDs are presented. The statistical significance of differences was determined by one-way ANOVA with a *post hoc* Dunnett’s multiple-comparison test (*n* = 4).

Previously, it has been shown that nsp1 proteins of SARS-CoV and SARS-CoV-2 efficiently inhibit cellular translation (10, 11), and their C-terminal fragments interact with the RNA channel of the 40S ribosomal subunit (11). The KH/AA mutation in nsp1 strongly affected its translation inhibitory function (11, 29). To further analyze the function of nsp1 in the development of translational shutoff, we transiently expressed the wt protein and its variants with some of the above-described mutations and used Pur labeling to evaluate changes in cellular translation. Proteins were expressed from plasmid DNAs as fusions with GFP to control the efficiency of transfection, and expression cassettes contained the fragment of 5′UTR of SARS-CoV-2 G RNA (nt 1 to 89) to promote more efficient translation of wt nsp1. Compared to the cells expressing GFP alone, those expressing wt nsp1 demonstrated a 10-fold decrease in Pur incorporation (Fig. 5A and B). As expected, the expression of nsp1 containing KH/AA mutations in the C terminus did not affect translation efficiency. However, mutations in the N-terminal structured domain or in the beginning of the intrinsically disordered C-terminal domain (G98D, R99L, K129E, and G130R) also strongly reduced the ability of nsp1 to inhibit cellular translation. These data suggest that the N-terminal folded domain is involved in the nsp1-mediated translation inhibition.

### Mutations in nsp1 rescue cellular translation and affect viral replication in cells that are competent in type I IFN induction and signaling.

**FIG 6:**
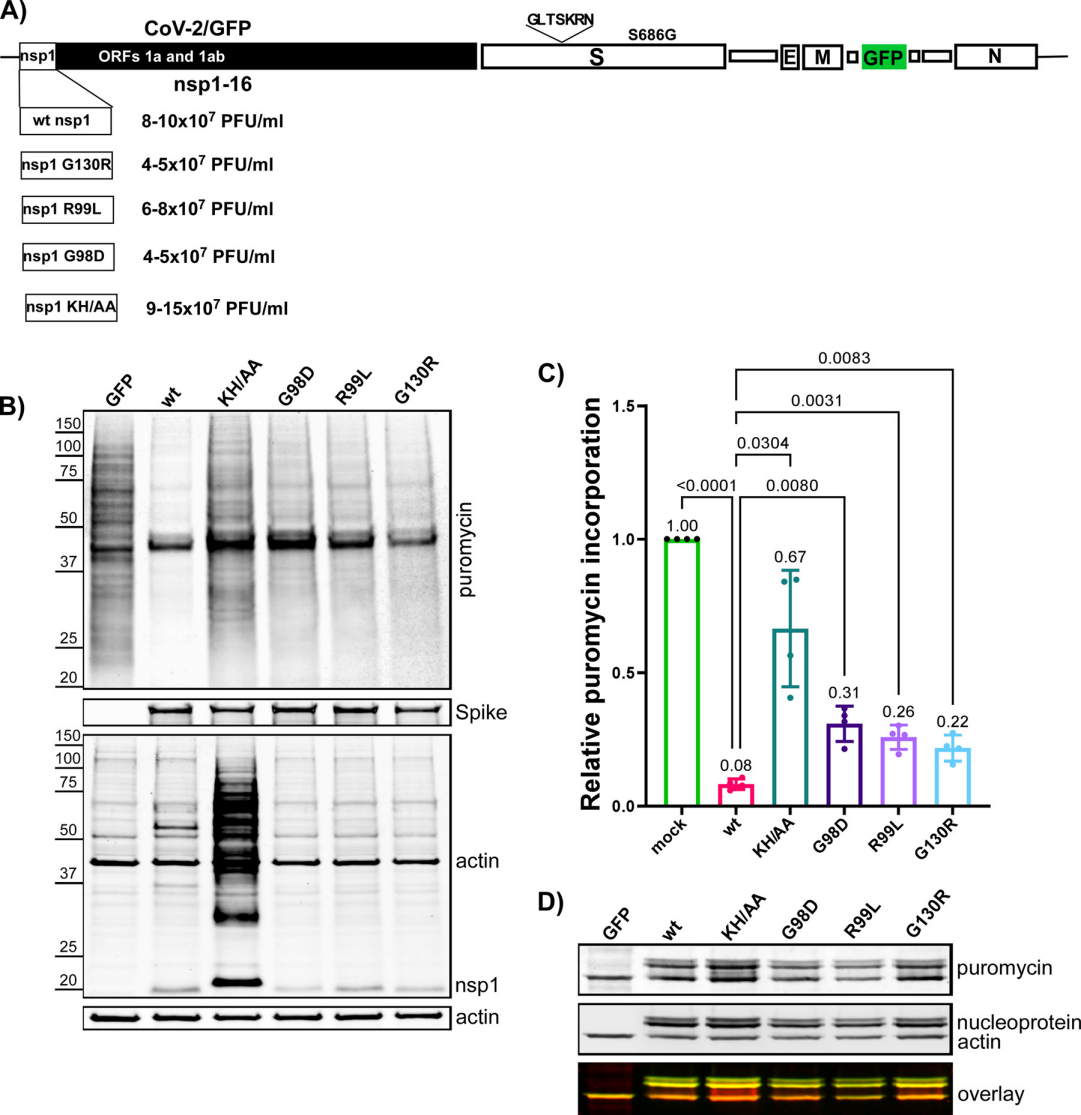
The nsp1-specific mutations have a negative effect on the abilities of SARS-CoV-2 to inhibit cellular translation, but they do not affect infectious viral titers. (A) The schematic presentation of SARS-CoV-2/GFP genome and the mutations introduced into nsp1 protein. Infectious titers of the rescued viruses were tested at 24 h of passage 2 on Vero/ACE2 cells with an MOI of 1 PFU/cell. (B) Vero/ACE2 cell were infected at an MOI of 2 PFU/cell. At 6 h p.i., media were replaced with that supplemented with 10 μg/mL of Pur. Cells were incubated for 15 min at 37°C, and harvested for Western blots. The membranes were processed with Pur-specific MAb and secondary Ab that were labeled with an infrared dye. The lower panels represent analysis of the samples with other indicated Abs. Images were acquired on LI-COR imager and processed using the manufacturer’s software. (C) Quantitation of the Pur-specific signals. The means and SDs are presented. The statistical significance of differences was determined by one-way ANOVA with a *post hoc* Dunnett’s multiple-comparison test (*n* = 3). (D) Western blot was processed using anti-Pur MAbs and antibodies specific to SARS-CoV-2 nucleoprotein and actin, followed by secondary antibodies with different fluorescent labels. Images were acquired using LI-COR imager.

Next, we tested the effects of selected nsp1-specific mutations on viral replication and virus-host interactions. Some of them (Fig. 6A) were introduced into infectious cDNA of SARS-CoV-2/GFP (CoV-2/GFP) (8, 30). The parental CoV-2/GFP variant contained a 7 aa long insertion after aa 214 in the S protein and a S686G mutation in the P1’ position of the furin cleavage cite. It was stable, infectious, and demonstrated no further evolution during at least two passages in Vero cells. The infectious cDNA clone also had ORF7a replaced by GFP to simplify tracking of the infection spread. The RNA genomes of the designed nsp1 mutants and parental CoV-2/GFP were synthesized *in vitro* by SP6 RNA polymerase and were transfected into BHK-21 cells that were stably expressing hACE2 and the N protein of SARS-CoV-2 (8, 30) transfected cells were then seeded onto Vero/ACE2 cells. All of the viruses, except the G98D mutant, were rescued with similar efficiencies, as indicated by complete CPE within 2 days post electroporation (PEP). The G98D mutant caused CPE only at day 4 PEP, suggesting that the mutation strongly affected its viability and that the recovered virus likely acquired adaptive mutation(s). After an additional passage on Vero/ACE2 cells, the viral stocks demonstrated similar infectious titers (Fig. 6A), and the plaques formed by the mutants were indistinguishable from those formed by the parental CoV-2/GFP (data not shown). This was a strong indication that nsp1 is not the only determinant of CPE development.

All the viruses were additionally analyzed via next generation sequencing (NGS), and all of them demonstrated the presence of mutations introduced into nsp1. No additional mutations were found in any of them, except the G98D mutant. In agreement with the low efficiency of rescue, the latter mutant acquired two additional mutations in the nsps (S167G in nsp1 and N386Y in nsp12), and these mutations likely compensated the negative effect of the G98D substitution on viral replication.

Next, we tested the effect of the mutations on translation inhibitory functions of the virus. Vero/ACE2 cells were infected with the indicated variants at an MOI of 2 PFU/cell, and the translation efficiencies were evaluated at 6 h p.i. by Pur incorporation (Fig. 6B and C). The KH/AA mutation in nsp1 had a strong negative effect on the virus-induced translational shutoff. The overall cellular translation remained at essentially the same level as that observed in mock-infected cells. Other mutants also demonstrated lower abilities to inhibit translation, but the effects were less prominent. Interestingly, we observed distinct bands generating an intensive Pur-specific signal. Two of them colocalized with those corresponding to the SARS-CoV-2 nucleoprotein (Fig. 6D). This observation suggested some abnormalities in the translation termination of the latter protein. In parallel, the infected Vero/ACE2 cells were additionally analyzed for accumulation of viral nonstructural nsp1 and structural proteins (Fig. 6B). There were no alterations in translation of structural S and N proteins (Fig. 6B and D). However, in cells infected with the KH/AA CoV-2/GFP mutant, nsp1 was detected at a higher concentration than in other samples (Fig. 6B). Moreover, a large fraction of the protein was present in the higher molecular weight bands. Their slower mobilities in the gel were suggestive of nsp1 monoubiquitination and polyubiquitination, but this possibility was not investigated further. The lysates of the cells that were infected with other mutants and the parental virus did not form such additional bands.

**FIG 7:**
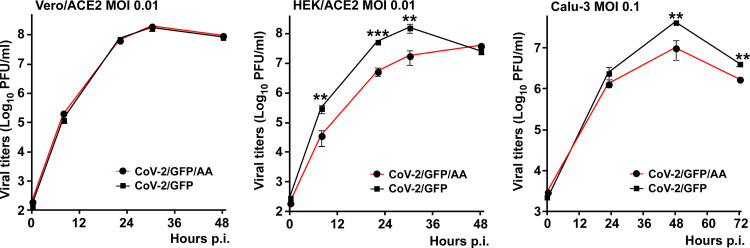
The KH/AA mutation in nsp1 has no negative effect on viral replication in Vero/ACE2 cells, but affects growth rates in the cells capable of type 1 IFN induction and signaling. The indicated cell lines were infected with wt SARS-CoV-2 and its KH/AA mutant at the same MOIs. At the indicated times p.i., media were replaced, and viral titers were determined by plaque assays on Vero/ACE2 cells. The experiment was repeated 3 times with highly reproducible results. The means and SD are presented. The statistical significance of differences was determined by Student's *t* test for each time point (*n* = 3).

Since the KH/AA mutant (CoV-2/GFP/AA) and the parental CoV-2/GFP viruses demonstrated the strongest difference in translation inhibition, we compared their replication rates in three different cell lines (Fig. 7). Their growth rates were indistinguishable in Vero/ACE2 cells, which are defective in type 1 IFN production. However, the nsp1 mutant replicated significantly less efficiently in Calu-3 and HEK/ACE2 cells, which are competent in type 1 IFN induction and signaling. Thus, the KH/AA mutation and likely other mutations in nsp1 affect SARS-CoV-2 replication *in vitro*, but this only occurs in type 1 IFN-competent cells.

### G98E mutation introduces local structural changes and prevents β5-strand formation.

**FIG 8:**
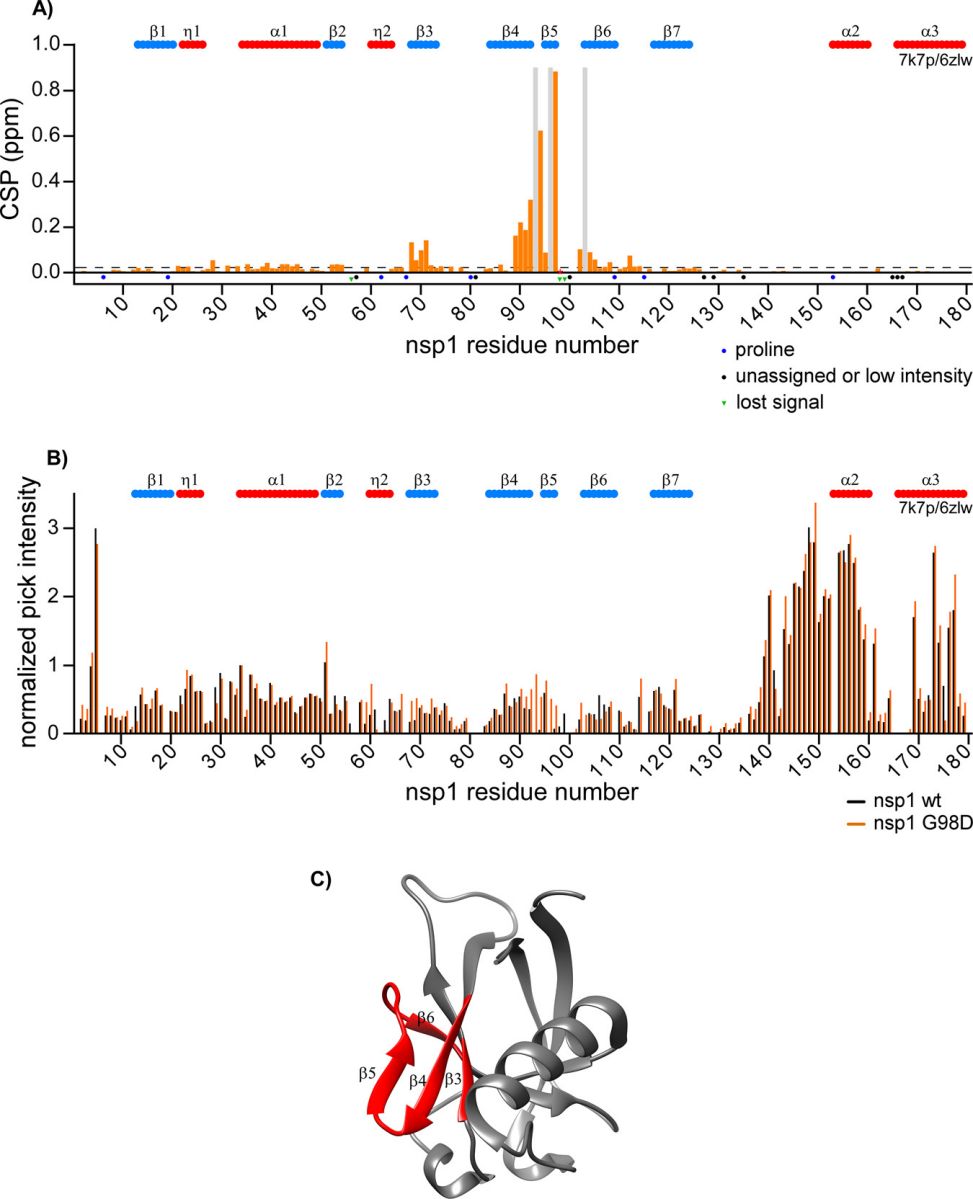
Comparison of wt SARS-CoV-2 nsp1 versus G98D mutant. (A) Chemical shift perturbations (CSP) of the amide bonds (^1^H and ^15^N nuclei) in the ^15^N-labeled wt nsp1 versus G98D mutant are presented as orange bars. The unassigned resonances and prolines are indicated by black and blue dots, respectively. The resonances observed only in the wt or in the mutant nsp1 are indicated by green dots and gray bars, respectively. The threshold of CSP values is indicated as a dashed line at the level of two standard deviations (2σ). (B) Normalized intensities of the cross peaks in ^1^H-^15^N TROSY spectra of wt nsp1 and G98D mutant are presented as black and orange bars, respectively. The previously determined elements of the secondary structures are presented on the tops of panels A and B. They were adapted from the X-ray data of the folded N-terminal domain of nsp1 (residues 10 to 124, PDB ID: 7k7p [20]) and from the CryoEM data of full-length nsp1 bound to the 40S ribosomal subunit (residues 153 to 179, PDB ID: 6zlw [11]). The β-strands and helices/turns are labeled in blue and red, respectively. (C) Crystal structure (PDB ID: 7k7p) of the folded domain of wt nsp1. The structural elements exhibiting CSP values in G98D mutant that are larger than the threshold are labeled in red.

Previously, we characterized wt SARS-CoV-2 nsp1 by high resolution NMR and reported the ^1^H, ^13^C and ^15^N resonance backbone assignments (22). In this new study, using the same approach, we assigned the backbone resonances of the nsp1 mutants and evaluated the effects of some mutations on the protein structure. First, we assessed the differences in chemical shifts (CS) and intensities of the cross peaks in the ^1^H-^15^N TROSY spectra between wt nsp1 and its G98D mutant (Fig. 8). The latter mutation demonstrated a deleterious effect on virus viability (see the previous section). Comparison of the normalized intensities of the amide ^1^H/^15^N cross peaks revealed no major differences between the spectra, with the exception of the region surrounding the mutation site (Fig. 8B). We also observed the resonances for residues 93, 96, and 103 in the G98D mutant spectra, which were not previously assigned in the wt counterpart. Intensities of other cross peaks in this region also strongly increased in the G98D spectra. These increases in signal intensities suggested either accelerated transition between different conformations or stabilization of a preferred conformation. No significant differences in the normalized intensities were observed for other regions. Thus, the introduced mutation had only local effect and did not globally disturb the structure of the entire folded N-terminal domain. Notably, the normalized intensities of cross peaks in the C-terminal domain (residues 130 to 180) were higher than the intensities of residues in the folded N-terminal region of both wt nsp1 and G98D mutant. This indicated an accelerated dynamic between different conformations in the N-terminal fragment, which is a common characteristic for intrinsically disordered domains, as we have previously described for the wt nsp1 (22).

Analysis of the chemical shift perturbations (CSP) revealed that the main structural differences between wt nsp1 and G98D mutant localized to two sites: the region surrounding the mutation site (residues 90 to 103 between the β4- and β6-strands) and in the β3-strand (residues 68 to 71) (Fig. 8A). Both regions are parts of the unique, six-stranded β barrel of the SARS-CoV-2 nsp1 (Fig. 8C).

**FIG 9:**
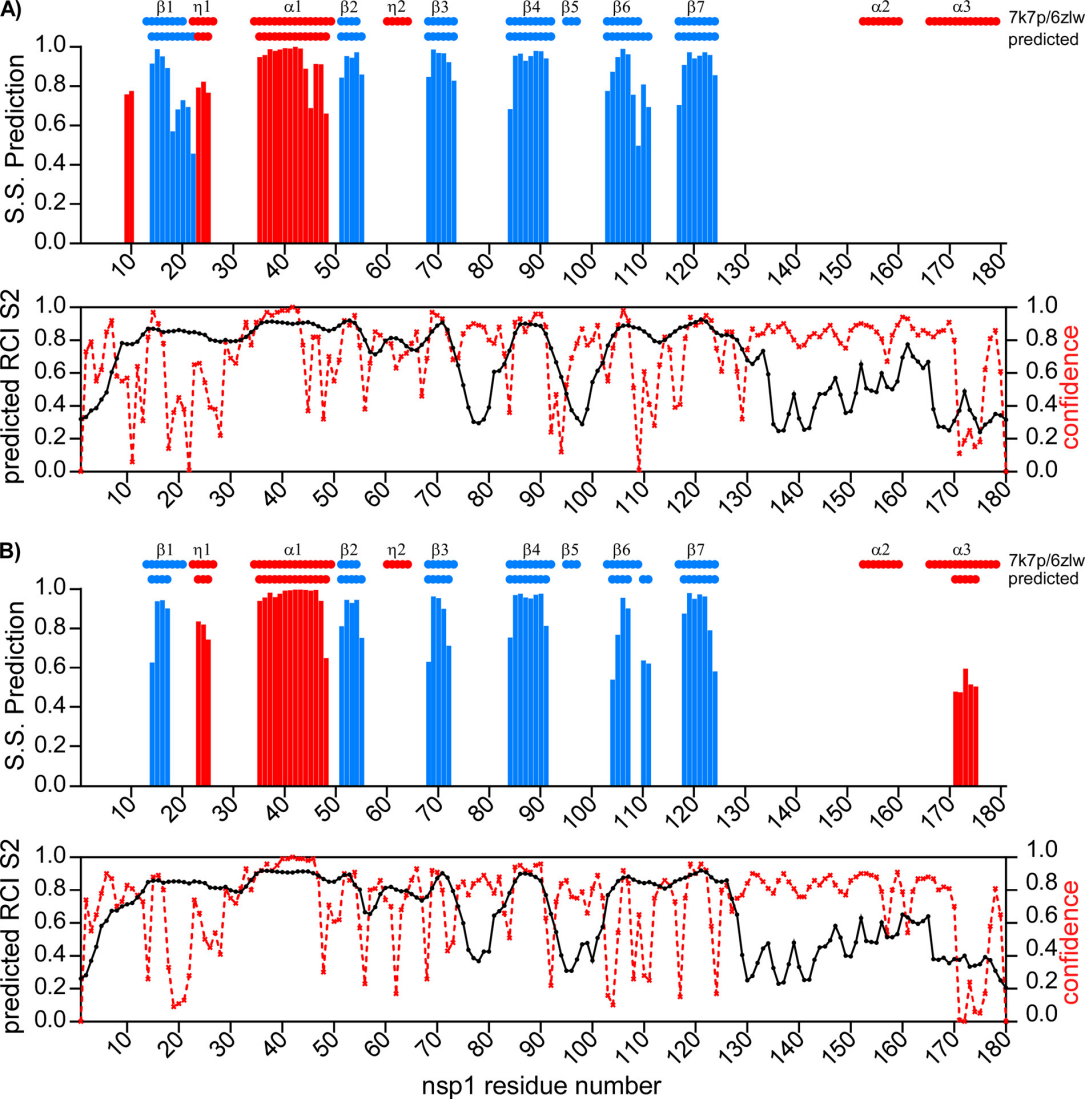
Secondary structures of wt nsp1 and G98D mutant. Secondary structure prediction (S.S. Prediction) index, RCI parameter, S2 (54), and confidence index were extracted from the CS of the ^1^H, ^13^C and ^15^N backbone resonances by TALOS-N (53) for wt nsp1 and G98D mutant (panels A and B, respectively). S.S. Prediction indexes versus the amino acid sequences are presented on the upper panels. Red and blue bars indicate α-helices/turns and β-strands, respectively. Predicted S2 and confidence index are presented on the lower panels, and they are shown by black and red lines, respectively. Schematic representations of the previously determined secondary structures are shown on the tops of the panels.

Next, we applied chemical shift data to predict the secondary structures of wt nsp1 and G98D mutant using TALOS-N and to compare them with those determined by X-ray (20) and cryo-electron microscopy (11) (Fig. 9). The structures of wt nsp1 and its G98D mutant were similar and contained the same number of β-strands and α-helices in the N-terminal folded domain. This was an indication that the mutation had no deleterious effect on the overall protein folding. However, a few subtle differences were detected. The β1-, β3-, and β6-strands in the G98D mutant were slightly shorter, despite the model-free order parameter, namely, S2, that was extracted by TALOS-N, remained in the same range for both proteins. In wt nsp1, the C-terminal fragment between residues 128 and 180 were predicted to have a random coil conformation with a high index of confidence between residues 128 and 170. However, based on our NMR data, the G98D mutant was predicted to have a short α-helix between residues 170 to 175 with a probability of approximately 60%, although the index of confidence for the prediction was low. This region undergoes a disorder-to-order transition upon binding to the 40S ribosomal subunit (11, 12).

The TALOS-N analysis of wt nsp1 and G98D mutant did not predict the presence of the β5-strand (amino acids 95 to 97) that had previously been identified in the crystal structure of wt SARS-CoV-2 nsp1 (PDB ID: 7k7p) and had been proposed to be specific for the latter protein (20, 21). Moreover, the predicted order parameters were gradually decreasing between residues 92 and 102, and they had the lowest values for residues 95 to 97. In the wt nsp1 spectrum, a majority of the residue cross peaks in this area were not present, with the exception of the cross peaks for the G98 and R99 residues (22). This was an indication of the dynamic mobility of this region and a slow exchange between distinct conformational assemblies. For G98D mutant, all of the cross peaks in this region were well-resolved and had high intensities. This allowed us to confirm the absence of hydrogen bonds between A90-Y97 and I95-L92 in the 3D NOESY spectrum of G98D mutant, which are required to be between the β5- and β4-strands.

**FIG 10:**
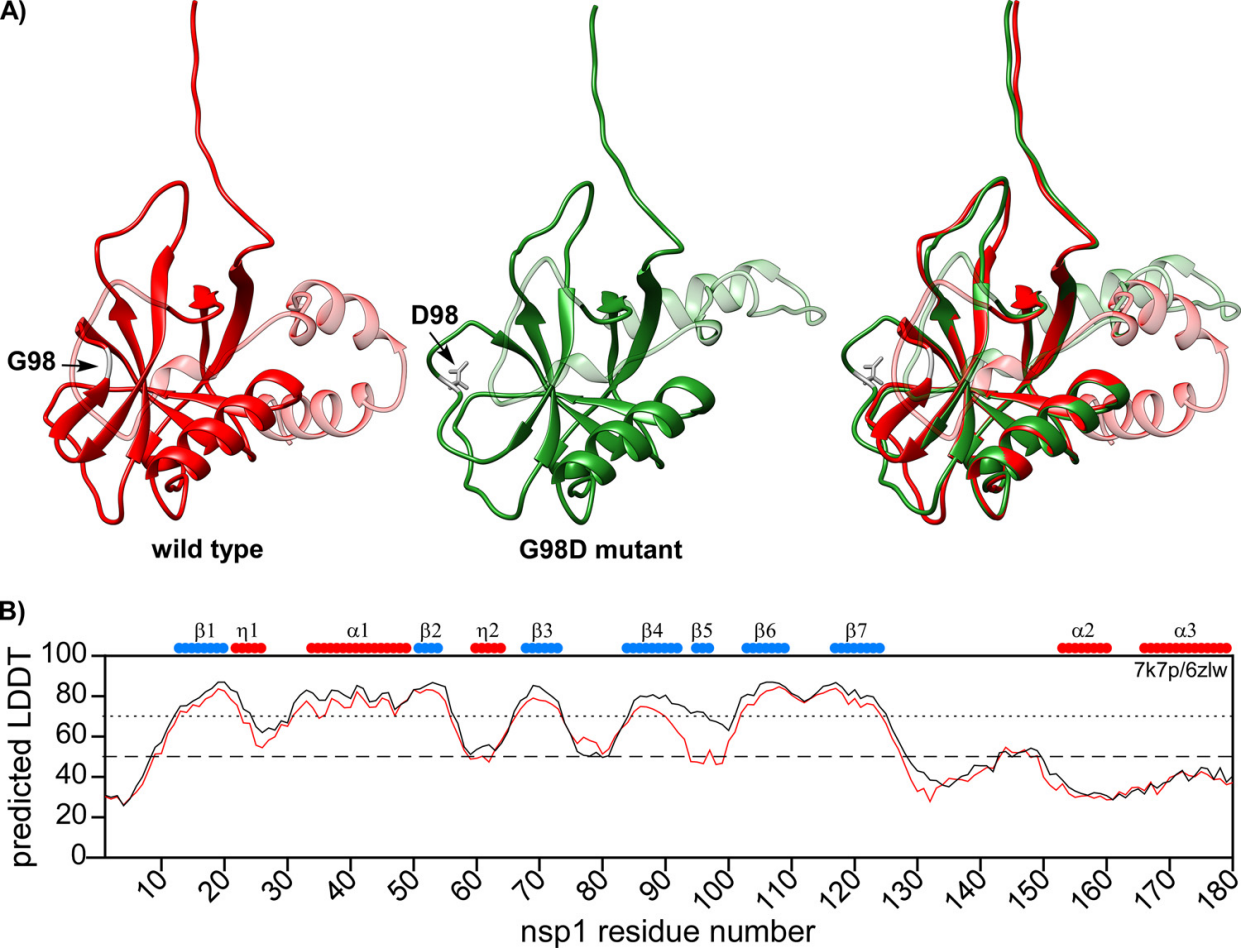
Predicted 3D structures of wt SARS-CoV-2 nsp1 and G98D mutant. (A) ColabFold v1.1 structure prediction for full-length wt nsp1 (red) and G98D mutant (green). The C-terminal disordered domains are presented as transparent. The sites of G98 and D98 are colored in gray. (B) The predicted LDDT plot. The wt nsp1 and G98D mutant are shown by black and red lines, respectively. Schematic representations of the previously determined secondary structures are shown on the top of the panel.

Finally, we applied ColabFold v1.1 to build the 3D structures of the wt and G98D mutant nsp1 proteins using the AlphaFold algorithm (Fig. 10) (31, 32). The overall structures of the folded N-terminal domains were similar, and the confidence of predictions, as determined by the predicted local distance difference test (pLDDT), was high for most of the secondary structures (pLDDT > 70). In correlation with our NMR data, the region between the β4 and β6 strands was predicted to form a loop in the G98D mutant with a pLDDT value of about 50%. The low pLDDT in the AlphaFold prediction suggested the presence of a disordered region (33). Thus, the model further confirmed that the G98D mutant does not have a β5-strand. However, the model for wt nsp1 predicted the presence of a β5-strand with high confidence. We also built the AlphaFold models for the nsp1 variants with the other mutations that were identified in this region: I95F, Q96L, and R99L (data not shown). The pLDDT values for I95F and R99L were similar to the pLDDT values of the wt nsp1 model. This suggests that the indicated mutations may have low destabilizing effects on the formation of the β5-strand and rather inhibit interactions with viral or host proteins. The pLDDT value for Q96L was lower than that of wt nsp1, suggesting a destabilizing effect of the mutation. Importantly, we used the original ColabFold v1.1 in our study. When we later attempted to build the models using ColabFold v2, with optimized algorithms and parameters, the G98D mutant structure was predicted to have a β5-strand. This discrepancy is in concordance with the recent reports that AlphaFold v2 poorly predicts the effects of point mutations and tends to utilize the available X-ray structures (34).

Taken together, our data suggest that the region between the β4- and β6-strands forms two structural assemblies that contain either a short β-strand or a flexible loop, and both conformations are in slow exchange in the wt protein. Introduction of the G98D mutation stabilizes the conformation with a flexible loop. Further molecular dynamics studies would allow us to better understand the contributions of different conformational assemblies. It would be interesting to identify whether any of the mutations that were found in this work would have effects on the stabilization of the β5-strand. The possibility of a connection between the β5-strand, nsp1 cytotoxicity, and viral pathogenesis calls for further investigation.

### The K129E mutation reveals two distinct structural assemblies of nsp1 and a potential interaction site between the N-terminal and C-terminal domains.

**FIG 11:**
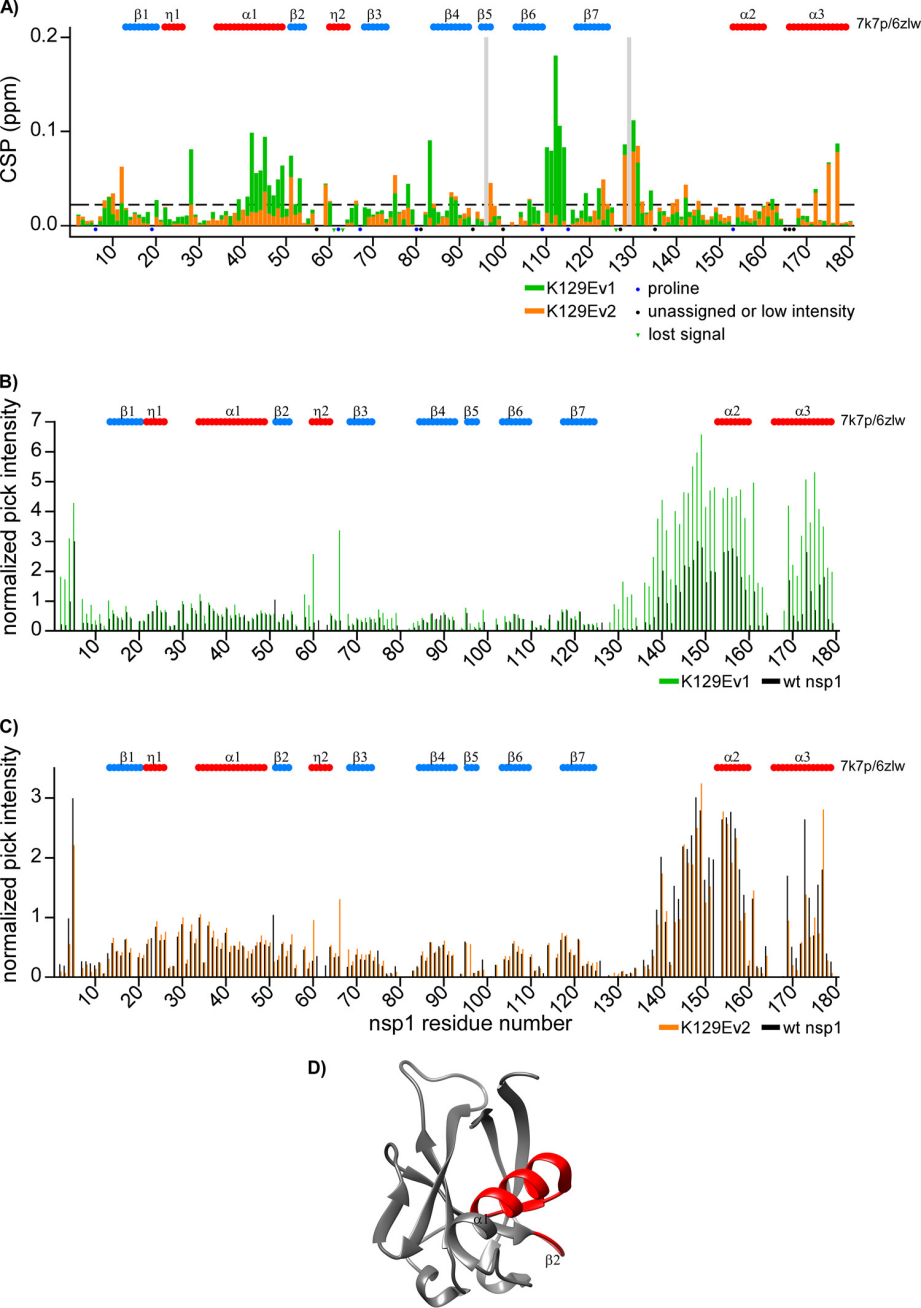
Comparison of wt SARS-CoV-2 nsp1 versus K129Ev1 (form 1) and K129Ev2 (form 2) mutants. (A) The chemical shift perturbations (CSP) of the amide bonds (^1^H and ^15^N nuclei) in the ^15^N-labeled wt nsp1 versus K129Ev1 (form 1) and K129Ev2 (form 2) nsp1 mutants are represented by green and orange bars, respectively. The unassigned resonances and prolines are indicated by black and blue dots, respectively. Resonances observed only in wt nsp1 or in K129E mutant are indicated by green dots and gray bars, respectively. The threshold of CSP is indicated as a dashed line at the level of two standard deviations (2σ). (B) Normalized intensities of the cross peaks in ^1^H-^15^N TROSY spectra of wt nsp1 and K129Ev1 (form 1) mutant are presented as black and green bars, respectively. (C) Normalized intensities of the cross peaks in ^1^H-^15^N TROSY spectra of wt nsp1 and K129Ev2 (form 2) mutant are presented as black and orange bars, respectively. The schematics of the previously determined secondary structures are presented on the tops of panels A to C, as described in Fig. 8. (D) Crystal structure (PDB ID: 7k7p) (20) of nsp1, with the structural elements of K129Ev1 (form 1) that have CSP values larger than the threshold are indicated in red.

The K129E mutation, which strongly affected the protein’s ability to cause translational shutoff (Fig. 5A), is located in the junction between the folded and intrinsically disordered domains of nsp1. While acquiring NMR spectra, we noticed that the mutant protein exhibited an unusual behavior. Within 2 to 3 days of data acquisition, the original ^1^H-^15^N TROSY spectra of the samples were noticeably changing. At the end, the protein became stable and allowed acquisition of more data. This suggested a sudden transition between two distinct conformational assemblies in solution, which we called the short-lived (K129Ev1) and long-lived or stable (K129Ev2). The analysis of the NMR spectra of both forms by TALOS-N revealed no changes in the secondary structure, compared to wt nsp1 (data not shown). However, strong differences were observed in the normalized intensities and CSPs (Fig. 11). The short-lived form of nsp1, K129Ev1, demonstrated the highest difference with the spectrum of the wt nsp1. The cross peak intensities of K129Ev1 increased in both intrinsically disordered regions, the N-terminal (residues 2 to 4), and the C-terminal (residues 128 to 179). However, the cross peak intensities of K129Ev2 were similar to those of its wt counterpart. For both forms, we detected that CSPs were in close proximity to the K129E point mutation (residues 128 to 131). There were no other changes in the K129Ev2 spectrum. However, the K129Ev1 spectrum contained two additional regions with CSPs: residues 39 to 53 in the α1-helix and residues 110 to 115 in the loop between the β6- and β7-strands within the N-terminal folded domain. The CS changes in these regions are not directly evident; they are located in the N-terminal folded domain, far away from the mutated residue, as shown on the structure (Fig. 11D). Previously, we observed two sets of amide ^1^H/^15^N cross peaks for residues 122 to 125 in the wt nsp1, which suggested the presence of two distinct conformational assemblies being detectable on the NMR time scale (22). The amide ^1^H/^15^N cross peaks for K129 were not assigned in the wt nsp1 spectra. The cross peak for E129 was assigned for both forms of the mutant and had a higher normalized intensity for K129Ev1. Note that we still observed double amide ^1^H/^15^N cross peaks for residues 122 to 125 in the K129E mutant.

**FIG 12:**
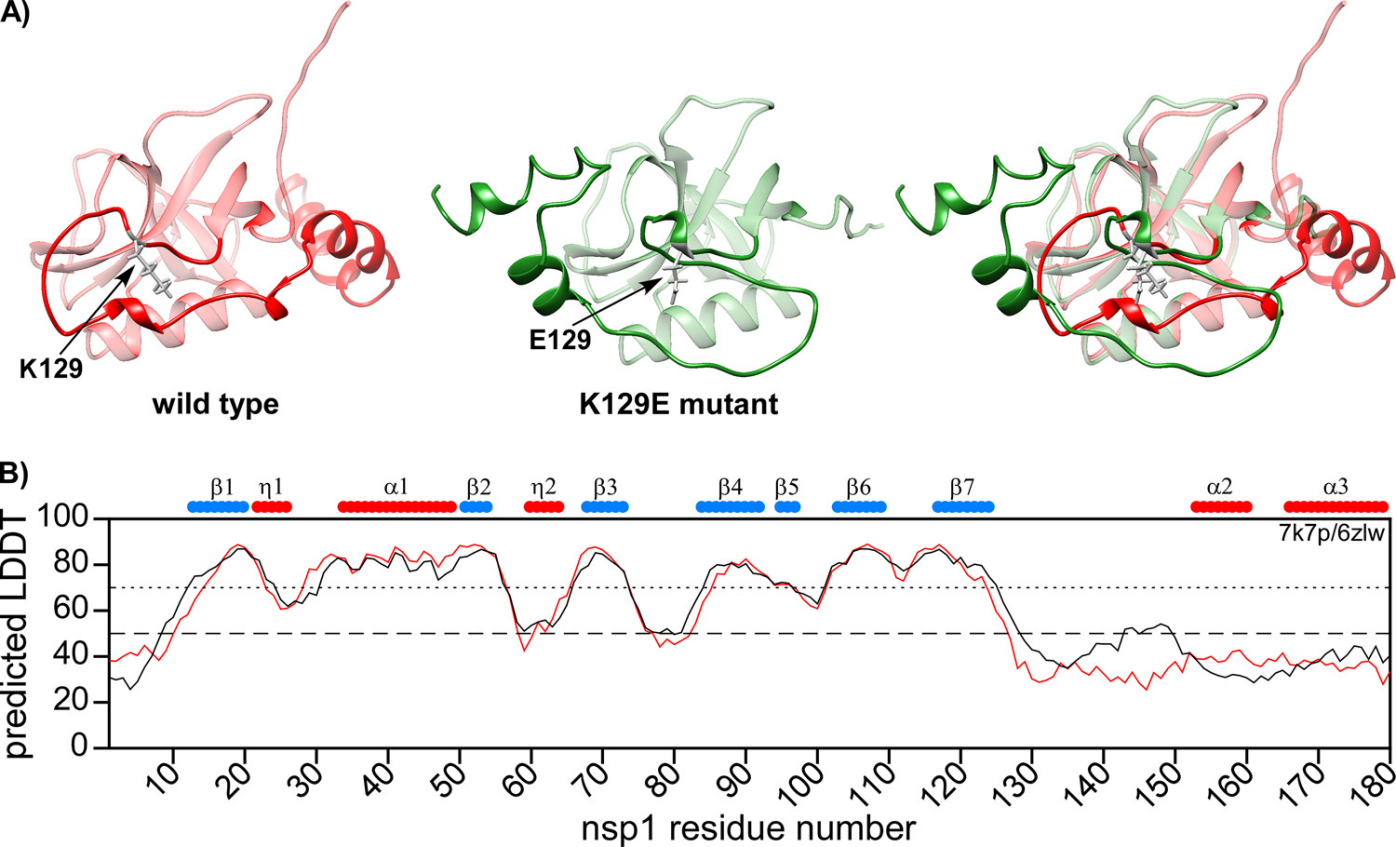
Predicted structures of wt SARS-CoV-2 nsp1 and K129E mutant. (A) ColabFold v1.1 structure prediction of the full-length wt nsp1 (red) and K129E mutant (green). The N-terminal folded domains are presented as transparent. The sites of K129 and E129 are colored in gray. (B) The predicted LDDT plot. Wt nsp1 and K129E mutant are represented by black and red lines, respectively. The schematic representations of the previously determined secondary structures are shown on the top of the panel.

The AlphaFold model of the K129E mutant did not reveal significant changes in the structure of the N-terminal folded domain (Fig. 12). However, the model showed the formation of a new 3_10_-helix at the site of the mutation. Therefore, the orientation of the C-terminal domain toward the N-terminal folded domain in K129E mutant differed from that of wt nsp1.

There are two important conclusions from these data. First, there is an interaction between the N-terminal domain and the C-terminal domain. Second, the flexibility of the region around K129 appears to be important for nsp1 function in the inhibition of cellular translation. We speculate that in the K129Ev1 form, the C-terminal domain is extended and does not interact with the folded domain. This would explain the increased intensities in the disordered region and CSPs in the folded domain. Then, there is an interplay between two different conformations of the mutant protein, and, ultimately, the system reaches equilibrium: the C-terminal domain folds on the N-terminal domain and interacts with the regions of residues 39 to 53 and 110 to 115. This hypothesis needs further experimental confirmation using other mutations in this site and molecular dynamics simulations.

## DISCUSSION

The nsp1 protein demonstrates a relatively low level of amino acid identity between the members of different CoV genera. However, despite the strong heterogeneity, it appears to exhibit similar functions among CoV species and is considered to be one of the major determinants of viral pathogenesis (10). It is well-established that the major function of nsp1 is global inhibition of cellular translation. Downregulation of cellular mRNA translation is utilized by many rapidly replicating RNA viruses for efficient inhibition of cellular antiviral response (25, 35). The nsp1-induced translational shutoff appears to also interfere with development of the antiviral response, and contributes to cell death during viral replication and ultimate development of CPE. In this new study, we utilized an unbiased approach to identify mutations in nsp1 that affect its translation inhibitory functions and make this protein noncytotoxic. These mutations accumulated in three different sites of nsp1. A large number of them were detected in the most C-terminal fragment of nsp1 (residues 152 to 180, region 1). Another cluster was found in the surface-exposed peptide of the N-terminal folded domain (residues 95 to 99, region 2). The third small cluster was in the junction between the N-terminal folded domain and the C-terminal intrinsically disordered domain (residues 129 to 130, region 3). Mutations in all three clusters abrogated nsp1-specific CPE. Importantly, they also strongly affected the inhibitory effect of nsp1 on cellular translation.

Region 1 (residues 152 to 180) has been shown to form two α-helices upon binding to the mRNA channel of the 40S ribosomal subunit. Accordingly, the K_164_H_165_/AA (KH/AA) mutation has been demonstrated to prevent this binding (11) and to make nsp1 incapable of inhibiting cellular translation (Fig. 6B). We have identified a wide range of other mutations in this region that have also made nsp1 noncytotoxic and have strongly affected inhibition of translation. Nevertheless, none of the mutations tested, including the previously described KH/AA, made the virus noncytopathic, although the KH/AA mutant demonstrated less efficient replication rates in type I IFN-competent cells. Surprisingly, we found that the KH/AA mutation strongly increased translation from viral genomic RNA (Fig. 6B) but had no effect on translation from subgenomic RNAs (at least those encoding spike and nucleoprotein). This mutation also had no effect on the translation of nsp1 from DNA cassettes containing truncated SARS-CoV-specific 5′ UTR that mimicked the leader of SG RNAs. This finding additionally suggests that the genomic 5′ UTR contains a translational enhancer or IRES, as was recently proposed (36). Its presence appears to provide an advantage for the translation of viral G RNA over cellular mRNAs at the early stages of viral replication. However, at later stages, the accumulation of wt nsp1, but not its KH/AA mutant, and the development of translational shutoff likely redirect translation from the genomic to subgenomic RNAs. Although the effects of genomic 5′UTR on translation in the presence of nsp1 have been extensively studied, the role of 5′UTR in the absence of translational shutoff needs further investigation. This finding also raises a caution against using viruses with the KH/AA mutation as vaccine candidates because of the more efficient production of the nonstructural viral proteins.

Region 2 (residues 95 to 99) is located between the β4- and β6-strands in the folded domain. In the selected mutants, four out of five amino acids in this region were found to be mutated, suggesting that this region is essential for inhibition of cellular translation by nsp1. All of the mutations made nsp1 noncytotoxic, and the two that were analyzed further, G98D and R99L, significantly reduced the nsp1-mediated translational shutoff. However, they did not completely restore cellular translation, as did the KH/AA substitution. This suggests that region 2 has a supporting function. The CryoEM structure of nsp1 bound to the ribosome had poor resolution of the folded domain (11, 12), and this could be explained by the existence of multiple bound states. Interestingly, in the structure of SARS-CoV nsp1, as determined by NMR, this region forms a flexible loop. However, the X-ray structure SARS-CoV-2 nsp1 demonstrated the presence of a short β-strand that is formed by residues 92 to 94 (20). It has been proposed that formation of the extra β-strand (β5) is unique for SARS-CoV-2 nsp1 and may be important for viral pathogenesis. Using NMR spectroscopy, we and others found that in solution, the β5-strand was not stably present in wt nsp1 of SARS-CoV-2 (22, 37). In addition, the absence of well-defined amide ^1^H/^15^N cross peaks for several residues in this region suggested the presence of several distinct conformations in slow exchange. Interestingly, in the noncytotoxic G98D mutant protein, this region was well-resolved in the NMR spectrum and clearly did not form a β-strand. The AlphaFold models further confirmed that nsp1 with the G98D mutation contained no β5-strand, but it was predicted for wt nsp1. Based on these data, we speculate that in solution, this region exists in at least two distinct conformations: open loop and β5-strand. Given that the G98D mutant had a functional defect, the ability to form the β5-strand may be essential for the inhibition of cellular translation. Since the position of nsp1 in the complex with the 40S ribosome subunit remains poorly defined by CryoEM, it is likely that the transition from an open loop to a β-strand may play a role in the regulation of nsp1 function. Recently, Mendez et al. also described the effect of an R99A mutation on SARS-CoV-2 nsp1 function (38). This protein mutant did not efficiently inhibit cellular translation and demonstrated reduced binding efficiency to the 80S ribosome. Taken together, these data support the hypothesis about the regulatory effect of conformational changes in the β5-strand on the inhibitory functions of nsp1. However, the analyses of the structure of this mutant in solution is needed. Of note, the X-ray structure for SARS-CoV nsp1 has not been determined. Thus, it is possible that in the latter protein, the above-described region has similar structural flexibility.

Interestingly, the G98D mutation also had a strong negative impact on virus replication, and only the variant with additional, adaptive mutations had been rescued. The first additional mutation was a S167G substitution in nsp1. It is located in region 1, but is conservative. The second mutation was found in viral RdRp, nsp12 (N338Y). This finding suggests that the structural flexibility of region 2 in nsp1 may be important for RNA replication. However, the possibility of an nsp1-nsp12 interaction needs additional experimental support.

The third cluster of mutations (residues 129 to 130) was found in the region between amino acids 123 to 130, which is identical in the SARS-CoV and SARS-CoV-2 nsp1 proteins. The double mutation K124A/K125A in SARS-CoV nsp1 has previously been shown to inhibit nsp1-associated exonuclease activity but not the translational shutoff (14). In SARS-CoV-2, the same mutation rescued cellular translation, but it had no effect on nsp1 exonuclease activity (38). In our screen, the mutations of K124 and K125 were not identified. Instead, we found mutations of K129E and G130R, suggesting that they likely had stronger impacts on the cytotoxicity of nsp1. Both also had strong negative effects on the translation inhibitory functions of the protein (Fig. 5). As was found for KH/AA, the G130R mutation had no detectable effect on viral titers or on its ability to cause CPE. We further analyzed K129E mutant by NMR spectroscopy and AlphaFold modeling. Previously, we found that some residues in region 123 to 130 in the wt nsp1 had two sets of amide ^1^H/^15^N cross peaks, which suggested the presence of two distinct conformational assemblies. Now, using NMR, we also found two distinct structural forms of K129E nsp1 mutant. The short-lived form demonstrated several cross peak perturbations in the N-terminal folded domain, which were indicative of interaction(s) between the folded and disordered domains of nsp1. The recently published results of the *in vivo* cross-linking experiments support these data (39). They also demonstrated an interaction between the N-terminal and C-terminal domains. Specifically, K125 was cross-linked with K11 or K47, and K129 was cross-linked with K47, K72, or K141. Thus, the flexibility of this region allows for the formation of different intermolecular interactions, and these appear to be essential for the function of nsp1.

### Conclusions.

(i) The NMR data and other results of this study demonstrate that nsp1 functions cannot be ascribed to a single domain. This protein has a dynamic structure, and its domains function cooperatively in regulating basic cellular functions. Neither the C-terminal α-helices nor the helices with the upstream-located disordered protein fragment can induce translational shutoff. Similarly, the structured N-terminal domain alone is also not cytotoxic and does not function as a translation inhibitor. (ii) Point mutations that make nsp1 noncytopathic were detected in the C-terminal helices, in a loop of the structured domain, and in the junction of the disordered and folded domains. (iii) The NMR-based data do not confirm the existence of a stable β5-strand that was proposed based on the X-ray structure. Most likely, in solution, this fragment is present in dynamic conformations that are required for the protein’s function in CPE development and in the regulation of protein expression and viral replication. (iv) Nsp1 is not the only determinant of virus-induced CPE. The identified mutations in this protein can make it noncytotoxic and incapable of inducing translational shutoff, but they do not have deleterious effects on cytopathogenicity of the virus.

## MATERIALS AND METHODS

### Cell cultures.

The BHK-21 cells were kindly provided by Paul Olivo (Washington University, St. Louis, MO). Calu-3 and VeroE6 cells were obtained from the American Type Culture Collection (Manassas, VA). Vero/ACE2 cells, which stably express hACE2, and BHK-21 cells expressing the nucleoprotein of SARS-CoV-2 and hACE2 have been described elsewhere (30). The BHK-21 and Vero E6 cells were maintained in alpha minimum essential medium supplemented with 10% fetal bovine serum (FBS) and vitamins. The Vero/ACE2 cells were maintained in the same medium supplemented with 20 μg/mL of blasticidin. Calu-3 cells were maintained in Dulbecco's modified Eagle’s medium supplemented with 10% (FBS).

### Plasmid constructs.

The cDNA of the SARS-CoV-2 (NC_045512.2) genome has been described elsewhere (8, 30). The mutations and deletions were introduced into nsp1 using standard PCR-based mutagenesis and cloning protocols. The sequences were verified by Sanger sequencing. The sequences encoding the full-length nsp1 and its derivatives were cloned into a wt (VEErep) and into noncytopathic (ncpVEErep) replicons of Venezuelan equine encephalitis virus (23) under the control of the SG promoter. The second promoter in ncpVEErep was driving the expression of the selectable marker, namely, Pac. The schematic presentations of the used constructs are shown in the figures. The plasmid encoding the helper RNA for the VEEV replicons has been described elsewhere (40). The expression plasmids encoded wt nsp1 and its mutants with Flag-GFP N-terminal tags, which were cloned under the control of the CMV promoter. The expression cassettes contained a partial 5′UTR sequence of the SARS-CoV-2 genome (ATTAAAGGTTTATACCTTCCCAGGTAACAAACCAACCAACTTTCGATCTCTTGTAGATCTGTTCTCTAAACGAACTTTAAAATCTGTGT) to facilitate nsp1 translation.

### Transfection of VEEV replicons and selection of Pur^R^ cells.

All of the plasmids were purified via ultracentrifugation in CsCl gradients. They were linearized by MluI, and replicons were synthesized *in vitro* using SP6 RNA polymerase in the presence of Cap analog, according to the protocol recommended by the manufacturer (New England Biolabs). The qualities and amounts of synthesized RNAs were tested via agarose gel electrophoresis in nondenaturing conditions. The analyses of the cytotoxicity and the selection of noncytopathic mutants were performed as described in our previous publications (24, 25, 27, 41, 42) (see also the figures for details). Briefly, 4 μg of the *in vitro* synthesized replicon RNAs encoding either nsp1, its fusions with GFP, or fusions of the designed protein mutants were electroporated into BHK-21 cells. Different numbers of the transfected cells were seeded into 100 mm dishes, and at approximately 16 h post RNA transfection, the media were supplemented with Pur at a concentration of 10 μg/mL. The numbers of Pur^R^ colonies were evaluated at 6 to 7 days posttransfection via staining with crystal violet. All of the experiments were reproducibly repeated 2 to 4 times. For the analysis of the mutations in nsp1 or in its GFP fusions, some of the colonies of Pur^R^ cells or GFP-positive clones of Pur^R^ cells were randomly selected, and the nsp1-coding sequences were amplified via RT-PCR. The synthesized fragments were purified and sequenced.

### Packaging of replicons into infectious virions.

For the packaging of VEEV replicons, their *in vitro*-synthesized RNAs were coelectroporated with helper RNAs into BHK-21 cells (40), and stocks were harvested at 24 h post transfection. To evaluate the efficiency of packaging, BHK-21 cells in 6-well Costar plates (5 × 10^5^ cell/well) were infected with serial 10-fold dilutions of packaged replicons and incubated at 37°C in complete media. The numbers of GFP-positive cells were evaluated on a fluorescence microscope at 16 h p.i.

### Rescue of recombinant viruses.

The cDNAs of the viral genomes were propagated as has been described elsewhere (8). After the *in vitro* ligation of 2 DNA fragments, RNAs were synthesized by SP6 RNA polymerase in the presence of the Cap analog and were electroporated into BHK-21 cells, which stably expressed hACE2 from the DNA construct integrated into cellular genome. These cells also expressed the SARS-CoV-2-specific N protein from the persistently replicating ncpVEEV replicon (ncpVEErep/N/Pac). Cells were seeded on the subconfluent monolayers of Vero/ACE2 cells in T75 flasks. Viruses were harvested at 2 days post-electroporation, after the cells became GFP-positive and developed profound CPE. The G98D mutant was harvested at day 4 post RNA electroporation. Aliquots of virus-containing media were used to infect monolayers of Vero/ACE2 cells, and stocks were harvested at approximately 24 h p.i.

Titers were determined via plaque assay on Vero/ACE2 cells. Cells were seeded in 6-well Costar plates (2.5 × 10^5^ cells/well) and, 4 h later, infected with serial 10-fold dilutions of viruses in PBS supplemented with 1% FBS. After incubation at 37°C for 30 min, the cells were covered with 2 mL of 0.5% agarose supplemented with DMEM and 3% FBS. At day 3 p.i., the cells were fixed with 7% formaldehyde and stained with crystal violet for the visualization of plaques.

### Viral growth rates.

Cells were seeded in 6-well Costar plates at a concentration of 5 × 10^5^ cells per well. After 4 h of incubation at 37°C, they were infected at the same MOI with the viruses encoding wt and mutated nsp1. The cells were washed twice with PBS and further incubated in the corresponding complete media. At the indicated times p.i., the media were replaced, and viral titers in the harvested samples were determined by plaque assay on Vero/ACE2 cells.

### Next generation sequencing (NGS) of viral genomes.

Harvested viral stocks were used for NGS. G RNAs were isolated from 0.2 mL of harvested samples using a Direct-zol RNA Miniprep Kit (Zymo Research), according to the manufacturer’s protocol. NGS was performed on a MiSeq Illumina in the UAB Heflin Center Genomics Core Facility, as has been described elsewhere (43). The raw sequence fastq files were aligned to the reference genome using BWA version 0.7.17-r1188. The aligned reads were then sorted with Picard version 2.9.2 SortSam.

### Analysis of translation in virus-infected cells.

Vero/ACE2 cells were infected with the recombinant viruses at an MOI of 2 PFU/cell for 30 min in PBS supplemented with 1% FBS. Then, they were incubated at 37°C in complete media. At 6 h p.i., the media were replaced with that supplemented with 10 μg/mL of Pur. Then, incubation continued for 15 min. The cells were harvested in PBS, pelleted via centrifugation, and lysed in standard protein loading buffer. The lysates were analyzed via Western blots using Pur-specific MAbs (Sigma) and infrared dye-labeled secondary Abs. The membranes were scanned on a LI-COR imager, and the data were processed, using the manufacturer’s software. The data were normalized to the amount of β-actin in the samples.

### Analysis of translation in transfected cells.

BHK-21 cells were transfected with the plasmids encoding Flag-GFP fusions of nsp1 proteins using Lipofectamine III (Invitrogen), according to the manufacturer’s protocol. At 18 h post transfection, the media were replaced with that supplemented with 10 μg/mL of Pur. Then, incubation continued for 15 min. Cells were harvested in PBS, pelleted via centrifugation, and lysed in standard protein loading buffer. The lysates were analyzed via Western blots using puromycin-specific MAbs (MABE343, Sigma) and infrared dye-labeled secondary Abs. Additional antibodies used: SARS-CoV-2 S2 (40590-T62, Sino Biological US Inc.), SARS-CoV-2 N (35-579, ProSci), SARS-CoV-2 nsp1 (GTX135612, GeneTex), GFP (600-145-215, Rockland), β-actin (66009-1-Ig, Proteintech). The membranes were scanned on a LI-COR imager, and the data were processed, using the manufacturer’s software Empiria Studia. The data were normalized to the amount of β-actin in the samples.

### NMR studies.

The plasmids for production of mutant proteins were constructed as described previously (22). The protein purification was performed as described elsewhere (22). The NMR experiments were acquired on Bruker Avance III spectrometers, operating at 14.1 T, that were equipped with a cryo-enhanced QCI-P probe at a temperature of 298 K. The backbone residues assignment of the wt SARS-CoV-2 nsp1 had previously been published (22). It was used as a starting point for the assignment of the corresponding resonances of the nsp1 mutants G99D and K29E in standard 3D TROSY triple resonance experiments. The iterative nonuniform sampling protocol (NUS) was comprised of HNCO, HNCA and HN(CO)CA, HN(CA)CO, as well as HN(CO)CACB and HNCACB experiments (44–47). A 25% sampling schedule was used for all other 3D spectra, yielding a total acquisition time of 153 h and an assignment time of approximately 1 week for the mutants. The summary of the performed experiments and the key parameters are presented in (22). The verification of the assignment of the mutated amino acids was also achieved by performing TROSY-type MUSIC experiments with a semiconstant time acquisition period in the indirect dimension. All of the TROSY-type MUSIC pulse sequences, as well as the setting details, have previously been described in full by us, elsewhere (48).

The H^α^ protons were assigned using a 3D HCACO sampling schedule that was comprised of 25% NUS. For the verification of the assigned H^α^ proton resonances and for the assessment of the NOE contacts, additional NOESY ^15^N-HSQC and NOESY-^13^C-HSQC (49–51) spectra were collected for every mutant. All of the acquisition data were processed in TopSpin4.1.3. The analyses were performed manually in CcpNmr Analysis 2.4.2 (52).

The chemical shifts (CS) of the ^1^HN, ^15^N, ^13^Cα, ^13^Cβ, ^13^C′, and H^α^ nuclei for every amino acid of the wt nsp1 and its G98D and K129E mutants were analyzed with the TALOS-N software (53) to extract the secondary structure prediction (S.S. Prediction) index, random coil index (RCI) (54) order parameter (S2), and confidence index. In cases of no chemical shift, TALOS-N uses a database of sequences to predict the secondary structure.

In the text and figures, the standard nomenclature for the amino acids of the carbon atoms was used, where ^13^Cα is the carbon next to the carbonyl group ^13^C′, and ^13^Cβ is the carbon next to ^13^Cα (55).

The 2D TROSY (^1^H–^15^N) spectra were used to extract the chemical shift perturbations (CSP) between the wt type SARS-CoV-2 nsp1 protein and its G98D and K129E mutants. The CSPs were obtained as the magnitude of the combined chemical shift deviations of the Δ^1^H and Δ^15^N nuclei (ppm), using the expression (ΔH1)2+(0.15ΔN15)22 with the normalizing coefficient of value 0.15 having been adopted from elsewhere (56). The thresholds of the CSP values are defined at the level of two standard deviations (2σ).

### AlphaFold prediction.

The structures of full-length wt nsp1 and the G98D and K129E mutants were predicted using ColabFold v1.1 (31, 32). The molecular visualizations were prepared using the UCSF Chimera package (57).
